# Soluble suppression of tumorigenicity 2 is a potential predictor of post-liver transplant renal outcomes

**DOI:** 10.1371/journal.pone.0293844

**Published:** 2023-11-02

**Authors:** Jong Joo Moon, Suk Kyun Hong, Yong Chul Kim, Su young Hong, YoungRok choi, Nam-Joon Yi, Kwang-Woong Lee, Seung Seok Han, Hajeong Lee, Dong Ki Kim, Yon Su Kim, Seung Hee Yang, Kyung-Suk Suh

**Affiliations:** 1 Seoul National University Biomedical Research Institute, Seoul, Korea; 2 Department of Surgery, Seoul National University College of Medicine, Seoul, Korea; 3 Division of Nephrology, Department of Internal Medicine, Seoul National University Hospital, Seoul, Korea; 4 Department of Internal Medicine, Seoul National University College of Medicine, Seoul, Korea; 5 Kidney Research Institute, Seoul National University Medical Research Center, Seoul, Korea; Indiana University School of Medicine, UNITED STATES

## Abstract

Acute kidney injury is considered an independent prognostic factor for mortality in patients with liver cirrhosis. Non-treated acute kidney injury can progress to hepatorenal syndrome with a poor prognosis. As suppression of tumorigenicity 2 (ST2) is a member of the interleukin-1 receptor family that aggravates inflammation and fibrotic changes in multiple organs, we measured soluble ST2 (sST2) level in the serum and urine of liver-transplant recipients at the time of transplantation. The serum sST2 level significantly increased in liver-transplant recipients with suppressed kidney function compared with that in recipients with normal function. In recipients with severely decreased liver function (model for end-stage liver disease score ≥ 30), the serum sST2 level was higher than that in recipients with preserved liver function (model for end-stage liver disease score ≤ 20, *P* = 0.028). The serum sST2 level in recipients with hepatorenal syndrome was higher than that in liver-transplant recipients without hepatorenal syndrome (*P* = 0.003). The serum sST2 level in patients with hepatorenal syndrome was higher than that in recipients without a history of acute kidney injury (*P* = 0.004). Recipients with hepatorenal syndrome and recovered kidney function showed higher sST2 levels than those who did not recover (*P* = 0.034). Collectively, an increase in the serum sST2 level reflects a decrease in both kidney and liver functions. Thus, measuring sST2 level at the time of liver transplantation can help predict renal outcomes.

## Introduction

Among patients hospitalized for decompensated liver cirrhosis (LC), acute kidney injury (AKI), a prevalent complication, is an independent prognostic factor for increased mortality and post-liver transplantation (LT) mortality [1, 2]. Acute kidney injury is associated with long-term mortality, a gradual decrease in kidney function, and a poor prognosis even after recovery from AKI [3]. Hepatorenal syndrome (HRS), a unique form of AKI, is a complication with a poor prognosis and high mortality. It indicates marked kidney function impairment in patients with advanced cirrhosis or acute liver failure [4]. In patients with LC, various factors such as bacterial infection (46%), hypovolemia (32%), HRS (13%), and other types of kidney diseases (9%) contribute to the development of AKI. Among these causes, HRS is associated with the lowest 90-day probability of survival (15%), and the median survival of patients with HRS is just 41 days [5]. Another study reported that the 90-day mortality rate of patients with HRS is 58% [6].

LT is the definitive treatment that improves long-term survival of patients with HRS [7]. However, deteriorated kidney function before transplantation could reduce survival and increase complications after transplantation [8, 9]. The 2–3-year survival rate of liver-transplant recipients (LTRs) without HRS is approximately 80%, although that of LTRs with HRS is approximately 60% [10, 11]. However, recent studies have revealed that LTRs with recovered kidney function from HRS have excellent renal prognosis and a 1-year survival similar to that of LTRs without a history of AKI [12]. Hence, there is a need for biomarkers to predict renal function recovery post-LT for long-term treatment planning and survival prediction in LTRs with HRS.

As the early detection of AKI is essential for improving treatment outcomes, biomarker candidates for AKI have been proposed, and these include cystatin C, kidney injury molecule-1, neutrophil gelatinase-associated lipocalin (NGAL), tissue inhibitor of metalloproteinase-2, and insulin-like growth factor (IGF)-binding protein-7 [13]. Among the proposed candidates, urine NGAL (uNGAL) and plasma cystatin C are potential AKI biomarkers in patients with decompensated LC [14]. In patients with LC, uNGAL could help distinguish the cause of AKI and predict mortality [15].

Suppressor of tumorigenicity 2 (ST2) is a member of the interleukin (IL)-1 receptor-like-1 family [16]. The two major forms of ST2 are the membrane-bound (STRL) and soluble (sST2) forms, which are produced by alternative splicing [17]. Interleukin-33, a member of the IL-1 family, binds to ST2L and activates the major pro-inflammatory transcription factor nuclear factor kappa B (NF-κB), which then translocates to the nucleus and binds to the DNA, exhibiting pro-inflammatory and fibrotic activities [18]. sST2 is secreted into the extracellular space, and elevated sST2 secretion is induced by inflammation, stress, and disease conditions, such as heart disease, exacerbation of asthma, eosinophilic pneumonia, sepsis, and trauma [19]. In liver diseases, the sST2 level reflects the extent of fibrotic liver changes in patients with chronic hepatitis B infection [20]. Among patients with alcoholic liver disease, a higher plasma sST2 level is observed in patients with poor prognostic scores, and it shows a positive relationship with the levels of inflammatory cytokines including IL-6 and IL-8 [21]. In the kidneys, an increase in the sST2 level is observed in patients with systemic lupus nephritis [22], advanced chronic kidney disease [23], and recurrent glomerulonephritis after kidney transplantation [24]. Therefore, sST2 is considered a potential prognostic predictor of kidney disease.

Here, we hypothesized that deteriorated liver and kidney functions affect the expression of sST2, which may be associated with the prognosis of renal recovery from HRS after LT. Therefore, we evaluated the sST2 level in urine and serum samples collected on the day of LT to test our hypothesis. We then compared the level of sST2 among LTR groups classified according to liver and kidney functions, history of HRS, and recovery from HRS. To demonstrate the potential of sST2 as a predictor of post-liver transplant renal outcomes, we also measured the urine level of NGAL, a known marker for renal damage, and compared it to the sST2 level.

## Materials and methods

### Ethics statement

This study was conducted in accordance with the tenets of the Declaration of Helsinki and approved by the Seoul National University Hospital Institutional Review Board (permit number: 2101-024-1185). Informed consent was obtained from all patients before using their blood and urine samples, which were collected and managed by the Seoul National University Hospital Human Biobank.

### Human samples

Serum and urine samples were obtained from LTRs and LT donors at Seoul National University Hospital from 2011 to 2020. Donor samples represented the normal control group. Using electronic medical records, patient data at the time of LT were collected, including age; sex; prothrombin time (PT); serum bilirubin, albumin, aspartate aminotransferase (AST), alanine transaminase (ALT), gamma-glutamyl transferase (GGT), ammonia, and creatinine levels; comorbidity; model for end-stage liver disease (MELD) score; Child-Turcotte-Pugh (CTP) score; and history of ascites, encephalopathy, and renal replacement therapy (RRT). Data of creatinine level at 3 months before and after LT were collected to evaluate baseline kidney function and recovery from HRS.

### Definitions

Hepatologists diagnosed LC and determined its cause. We determined HRS-AKI according to the recommendations of the International Club of Ascites using the following criteria: 1) absolute increase in serum Cr level ≥ 0.3mg/dL within 48 h or 2) percent increase in serum Cr level ≥ 50% using the last available value within 3 months as the baseline value [25]. The diagnosis and severity determination of AKI in patients with HRS were made using the Kidney Disease: Improving Global Outcomes (KDIGO) AKI guidelines by nephrologists. To exclude postoperative AKI, we identified patients with HRS among those who developed AKI before LT. We divided LTRs with HRS-AKI into two groups: recovery and non-recovery. Recovery of kidney function after LT was evaluated according to serum creatinine level 90 days after LT. Non-recovery of HRS after transplantation was defined as an elevated serum creatinine level of over 1.3 mg/dL and increased serum creatinine level by more than 0.3 mg/dL compared to the baseline creatinine level. Patients who maintained RRT were also included in the non-recovery group.

### Enzyme-linked immunosorbent assay

We assessed the levels of ST2 and NGAL in the serum and urine samples collected at the time of LT using a commercial enzyme-linked immunosorbent assay (ELISA) kit (R&D Systems, Minneapolis, MN, USA), per the manufacturer’s instructions.

### Statistical analysis

Statistical analyses were performed using GraphPad Prism 9 (GraphPad Software, San Diego, CA, USA). Student’s *t*-test was used for comparison between groups. Mann–Whitney U test was used to compare the levels of sST2 and uNGAL between groups. Categorical variables were compared using the chi-squared test. The odds ratios (ORs) and confidence intervals (CIs) for renal recovery were analyzed using a logistic regression analysis. The results are presented as mean and standard error. Statistical significance was set at *P* < 0.05.

## Results

### Serum sST2 level reflects liver and kidney functions in LTRs

**Fig 1:**
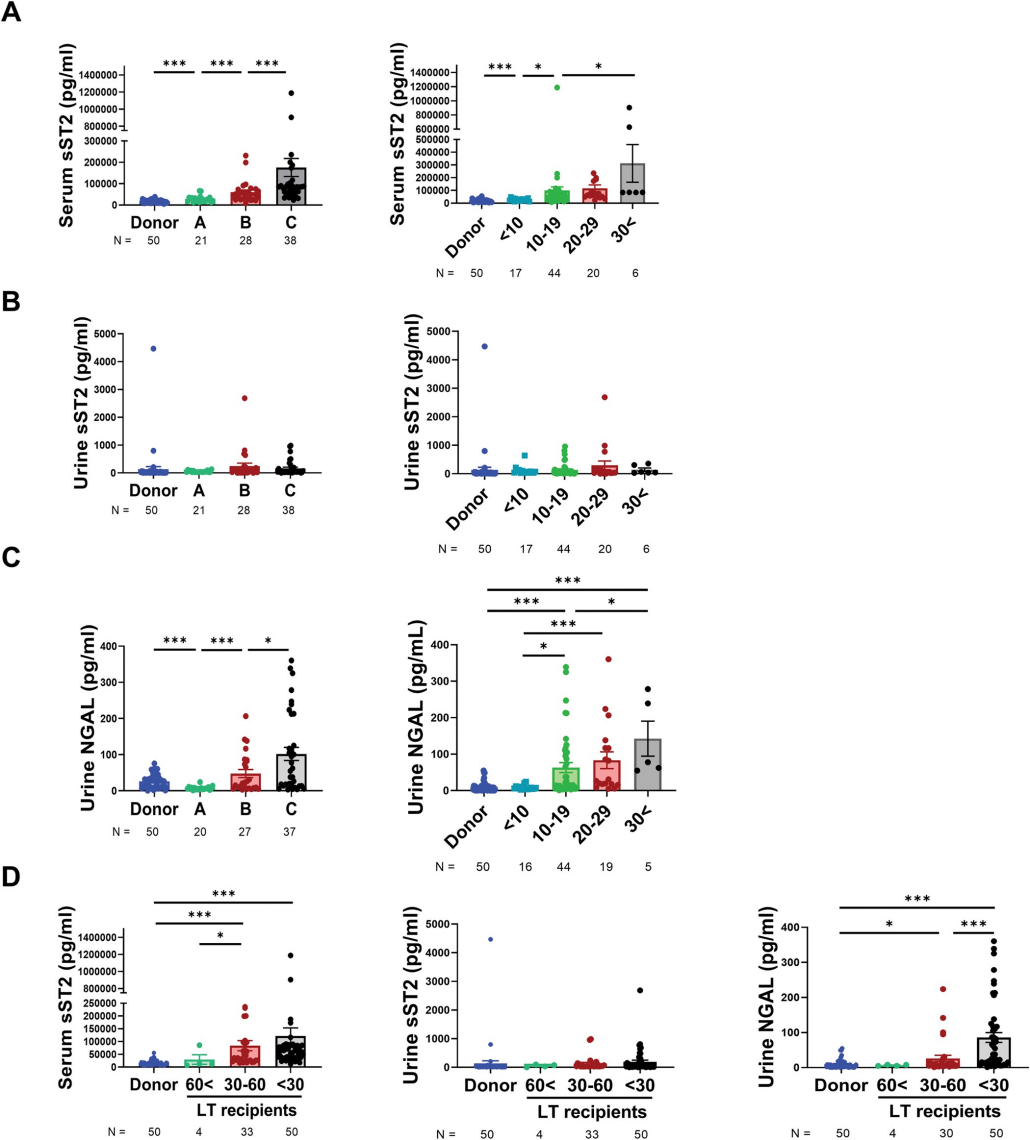
Increased serum sST2 and uNGAL levels reflect deteriorated liver and kidney functions. (A) Serum sST2 level was elevated in LTRs with deteriorated liver function. (B) Urine sST2 level exhibited no differences according to liver function. (C) uNGAL level increased with CTP or MELD scores. (D) Liver-transplant recipients were stratified into three groups based on the sST2 and uNGAL levels using the estimated glomerular filtration rate. Mann–Whitney U test was used to compare the levels of sST2 and uNGAL between two groups. *P < 0.05, **P < 0.01, ***P < 0.001.

To determine the effect of deteriorated liver function on the expression of sST2, the level of sST2 was measured in the serum and urine samples from LTRs and donors. The liver function was evaluated using the CTP and MELD scores. In this study, donors were considered healthy. An elevation in serum sST2 level was observed with a decrease in liver function, and it was the highest in the CTP class C group and LTRs with the highest MELD score (**Fig 1A**). In the urine, the sST2 level exhibited no pattern according to liver function (**Fig 1B**). The uNGAL level increased in the CTP class C group, showing a change pattern similar to that of the serum sST2 level (**Fig 1C**). Increased serum sST2 levels were observed in various laboratory and clinical assessments that reflected deteriorated liver function, such as high bilirubin, ammonia, AST, and ALT levels; high PT international normalized ratios (INRs); high MELD scores; and low albumin levels (**S1 Fig**). We stratified the LTRs into three groups to determine the effect of kidney function on sST2 level. The donor group had the lowest serum sST2 level, and LTRs with normal kidney function (estimated glomerular filtration rate (eGFR) > 60 mL/min/1.73 m^2^) also exhibited a lower serum sST2 level than LTRs with moderate to severely decreased kidney function (eGFR < 60 mL/min/1.73 m^2^; **Fig 1D, left panel**). The urine sST2 level did not significantly differ among the groups, whereas the uNGAL level in the severely decreased kidney function group significantly increased (**Fig 1D, middle and right panels**).

### LTRs with HRS have a higher serum sST2 level

The baseline patient characteristics are presented in **Table 1**. The HRS group showed high MELD and CTP scores and worsened laboratory test results such as high white blood cell counts; high bilirubin, AST, ALT, creatinine, and serum sST2 levels; and prolonged PT. There were no significant differences in age, body mass index, comorbidity, hepatocellular carcinoma, or urine sST2 level.

**Table 1:** Baseline characteristics of patients at the time of liver transplantation.

Variable	Donor	LT without HRS	LT with HRS	HRS vs. Non-HRS
	**(n = 50)**	**(n = 56)**	**(n = 31)**	***P***
Age (y)	32.87 ± 11.19	58.28 ± 10.52	54.16 ± 10.36	0.939
Sex (male, %)	58.00	58.93	70.97	0.265
BMI (kg/m^2^)	23.25 ± 3.20	23.25 ± 3.07	23.05 ± 5.40	0.818
MELD score		13.39 ± 4.77	23.87 ± 8.65	<0.001
CTP Score		7.96 ± 2.39	10.50 ± 2.08	<0.001
Cause of LC				
HBV (%)		35.71	25.81	0.898
HCV (%)		3.57	6.45	0.377
Alcohol consumption (%)		41.07	48.39	0.434
Others (%)		19.64	19.35	0.001
Comorbidity				
DM (%)		30.36	35.48	0.624
Hypertension (%)		3.57	9.68	0.258
Presence of HCC (%)		37.5	19.35	0.666
Laboratory test (serum)				
WBC (1,000/mL)	6.54 ± 1.86	4.34 ± 2.26	9.26 ± 6.51	<0.001
Hemoglobin (g/dL)	14.17 ± 1.47	10.28 ± 2.08	8.98 ± 1.77	0.0519
Platelet (1,000/mL)	243.5 ± 52.41	81.64 ± 49.40	69.09 ± 37.58	0.421
Bilirubin (mg/dL)	0.52 ± 0.25	2.86 ± 3.17	14.67 ± 15.33	<0.001
Albumin (g/dL)	4.54 ± 0.30	2.1 ± 0.58	2.88 ± 0.79	0.004
PT (INR)	1.00 ± 0.07	1.33 ± 0.29	2.15 ± 1.27	<0.001
Na (mmol/L)	141.2 ± 1.54	138.9 ± 4.11	134.0 ± 5.88	<0.001
Creatinine (mg/dL)	0.82 ± 0.16	1.01 ± 0.99	3.25 ± 1.86	<0.001
AST (IU/L)	16.82 ± 3.70	37.44 ± 17.89	66.66 ± 62.12	0.002
ALT (IU/L)	16.86 ± 8.27	25.36 ± 18.56	40.03 ± 47.10	0.042
GGT (IU/L)	20.88 ± 11.25	64.67 ± 87.02	65.06 ± 76.30	0.983
Serum ST2 (pg/mL)	16,788 ± 6,824	50,391 ± 52,702	176,381 ± 293,153	0.026
Urine ST2 (pg/mL)	136.5 ± 641.9	166.2 ± 412.3	260.7 ± 627.7	0.408

Values are presented as mean ± SD and % for categorical variables. Abbreviations: ALT, alanine transaminase; AST, aspartate aminotransferase; BMI, body mass index; CTP, Child-Turcotte-Pugh; DM, diabetes mellitus; GGT, gamma-glutamyl transferase; HBV, hepatitis B virus; HCC, hepatocellular carcinoma; HCV, hepatitis C virus; HRS, hepatorenal syndrome; INR, international normalized ratio; LT, liver transplantation; MELD, model for end-stage liver disease; PT, prothrombin time; WBC, white blood cell. Mann–Whitney U test was used to compare the levels of sST2 between HRS and non-HRS groups.

**Fig 2:**
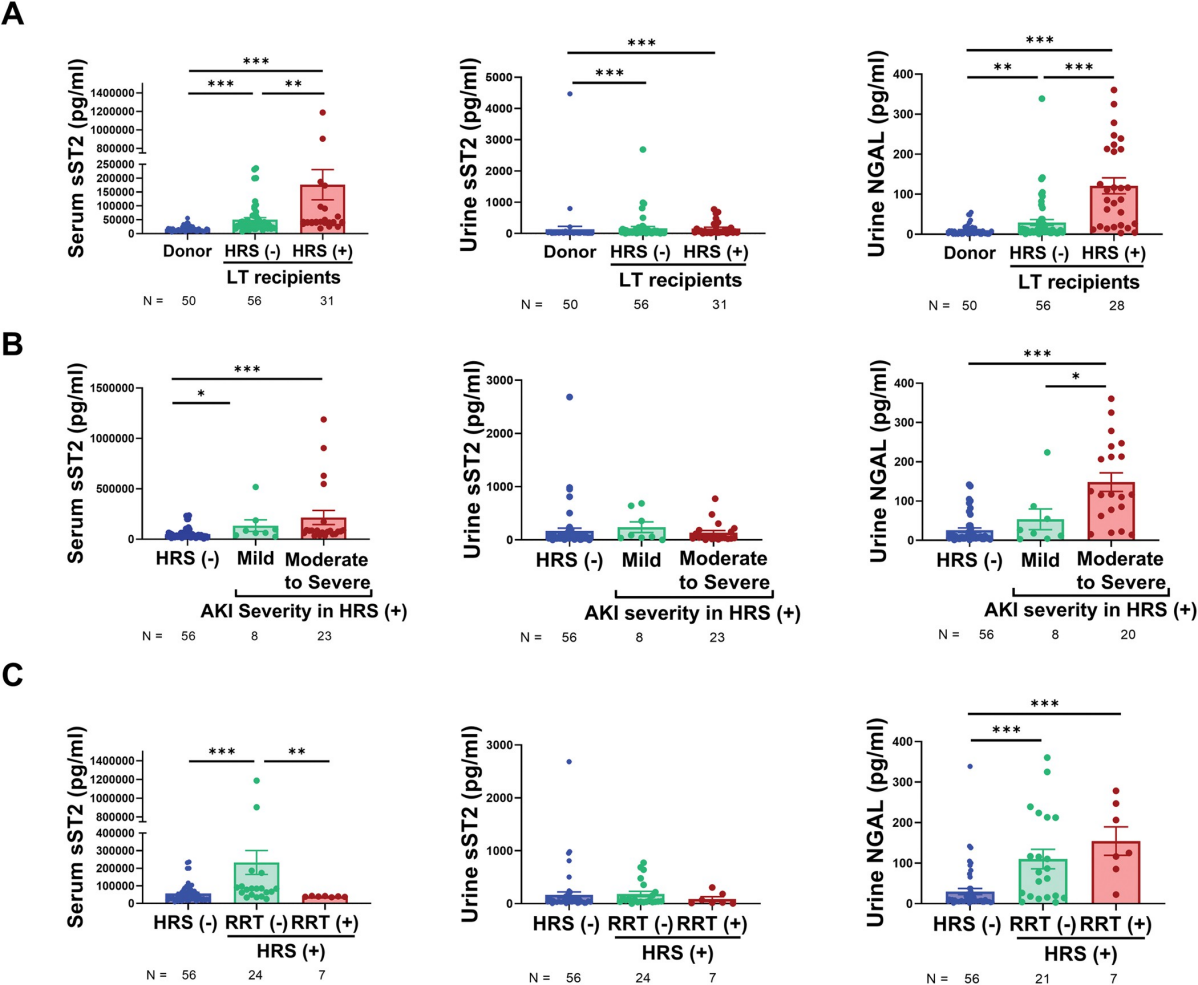
Liver-transplant recipients (LTRs) were classified based on the sST2 and uNGAL levels according to the history of HRS, AKI severity, and history of RRT. (A) Elevated sST2 and uNGAL levels were observed in LTRs with a history of HRS. (B) Increased sST2 and uNGAL reflected AKI severity in HRS. (C) LTRs were classified based on the sST2 and uNGAL levels using RRT history. Mann–Whitney U test was used to compare the levels of sST2 and uNGAL between two groups *P < 0.05, **P < 0.01, ***P < 0.001.

The serum sST2 level in the LTRs with or without HRS was higher than that in the donors. The HRS group showed significantly elevated serum sST2 level compared to the group without HRS (**Fig 2A, left panel**). The urine sST2 level was not significantly different between the groups (**Fig 2A, middle panel**). In contrast, the uNGAL level differed between the groups. The highest uNGAL level was observed in the HRS group (**Fig 2A, right panel**).

To determine the severity of AKI based on the sST2 level in LTRs with HRS, recipients with HRS were classified into two groups according to AKI severity. The serum sST2 level was low in donors and LTRs without AKI (**Fig 2A and 2B**). In the presence of mild-to-moderate AKI, the serum sST2 level increased, and it was the highest in LTRs with moderate-to-severe AKI (**Fig 2B, left panel**). There were no significant differences in the urine sST2 level among LTRs (**Fig 2B, middle panel**), whereas the uNGAL level increased with the severity of AKI (**Fig 2B, right panel**). The HRS group was divided into two groups: with and without a history of RRT. In patients with HRS without a history of RRT, the serum sST2 level increased compared with that in patients without a history of RRT (**Fig 2C, left panel**), although no significant differences were observed in the urine sST2 level between these groups (**Fig 2C, middle panel**). The uNGAL level was the highest in the RRT group (**Fig 2C, right panel**).

### Recovery from HRS could be predicted using the serum sST2 level in LTRs

Three months after LT, recovery from HRS was determined based on level of creatinine and a history of maintaining RRT. We classified patients with HRS into the recovery group if the creatinine level at 3 months after LT did not increase by more than 0.3 mg/dL above the baseline creatinine level. The non-recovery group consisted of patients with HRS whose creatinine level increased by more than 0.3 mg/dL compared to the baseline creatinine level or those who were continuing dialysis. No significant differences were observed in the baseline creatinine level and MELD score between the recovery and non-recovery groups (**Table 2**).

**Table 2:** Serum and urine levels of sST2 and NGAL.

	HRS (-) (n = 56)	HRS (+) (n = 31)		Recovery vs. Non-recovery	HRS (-) vs. Recovery	HRS (-) vs. Non-recovery
		Recovery (n = 16)	Non-recovery (n = 15)	*P*	*P*	*P*
Baseline Cr (mg/dL)	0.84 ± 0.27	0.94 ± 0.23	1.11 ± 0.37	0.165	0.211	0.005
MELD score	13.63 ± 4.86	25.09 ± 9.57	22.23 ± 8.55	0.410	<0.001	<0.001
CTP score	7.98 ± 2.40	11.19 ± 1.87	9.62 ± 2.14	0.440	<0.001	0.028
Serum sST2 (pg/mL)	56,036 ± 52,409	297,560 ± 354,810	66,554 ± 20,799	<0.001	<0.001	0.951
Urine sST2 (pg/mL)	165.8 ±412.5	187.7 ± 248.1	125.6± 182.2	0.767	0.701	0.798
Urine NGAL (pg/mL)	28.45 ± 63.09	113.4 ±109.5	130.1 ± 105.0	0.525	0.770	0.115

Values are presented as mean ± SD. Abbreviations: Cr, creatinine; CTP, Child-Turcotte-Pugh; HRS, hepatorenal syndrome; MELD, model for end-stage liver disease; NGAL, neutrophil gelatinase-associated lipocalin; sST2, soluble suppression of tumorigenicity 2 protein. Mann–Whitney U test was used to compare the levels of sST2 and uNGAL between two groups.

Renal recovery was determined using serum creatinine level at 3 months after LT. The serum sST2 level in the recovery group on the transplantation day significantly increased compared with that in the non-recovery group, whereas the urine sST2 and NGAL levels showed no significant differences between the groups. The uNGAL level on the day of LT was the highest in the non-recovery group but showed no significant difference (**Table 2**) compared with that in the recovery group.

To assess potential correlations or associations between variables and renal recovery at 3 months after LT, we conducted a logistic regression analysis. The LTRs with HRS were divided into two groups using median as a cut off value (serum sST2, 41,828.472 pg/mL; uNGAL, 113.151 pg/mL; MELD score, 21.190; CTP score, 11). The range of serum sST2 level was 18,755.34–41,725.32 pg/mL for the lower group (n = 15) and ≥41,828.472 pg/mL for the higher group (n = 16). In the logistic regression analysis with no adjustment, HRS patients with a higher serum sST2 level demonstrated higher renal recovery at 3 months after LT. HRS patients with a higher CTP score showed a lower tendency for renal recovery after LT. The serum sST2 level constantly showed significant differences even after adjusting several factors (**Table 3**).

**Table 3:** Odds ratios of renal recovery at 3 months after liver transplantation according to several factors.

	Crude			Adjusted		
	OR	95% CI	P	Adjusted OR	95% CI	P
Age	0.998	0.932–1.069	0.960	1.060	0.933–1.205	0.372
Sex (Men)	1.091	0.218–5.454	0.916	0.473	0.029–7.801	0.601
Cause of LT						
Viral (HBV, HCV)						
Alcohol consumption	0.914	0.174–4.811	0.916	0.186	0.007–5.096	0.319
Others	0.800	0.101–6.347	0.833	1.917	0.036–101.676	0.748
History of RRT	2.889	0.568–14.682	0.201	0.148	0.003–8.370	0.353
Presence of HCC	0.500	0.041–6.166	0.589	0.019	0.000–6.179	0.179
sST2 (Higher group, 1,000 pg/mL)	17.333	2.916–103.021	0.002	84.584	1.808–3957.940	0.024
uNGAL (Higher group, pg/mL)	1.143	0.279–4.683	0.853	5.248	0.209–131.501	0.313
MELD score (higher group)	0.400	0.094–1.699	0.214	1.194	0.086–16.657	0.895
CTP score (higher group)	0.167	0.035–0.793	0.024	0.153	0.006–3.782	0.251

Adjusted factors including age, sex, cause of LT (viral or alcohol), history of RRT, presence of HCC, sST2, uNGAL, MELD score, and CTP score. Abbreviations: OR, odds ratio; CI, confidence interval; HBV, hepatitis B virus; HCV, hepatitis C virus; RRT, renal replacement therapy; HCC, hepatocellular carcinoma; sST2, soluble suppression of tumorigenicity 2 protein; NGAL, neutrophil gelatinase-associated lipocalin; CTP, Child-Turcotte-Pugh; MELD, model for end-stage liver disease. The serum sST2 and uNGAL levels on the day of LT exhibited a weak correlation (*P* < 0.0001, R^2^ = 0.221).

## Discussion

Our study demonstrated elevated serum sST2 level in LTRs with deteriorated liver or kidney function. The serum sST2 level also reflects AKI severity as it was higher in patients with HRS than in those without HRS. However, significantly elevated serum sST2 level was observed in the recovered group compared with that in the non-HRS and non-recovered HRS group.

We measured the serum and urine sST2 levels and compared them to the uNGAL level. Several studies have shown the utility of uNGAL as a biomarker for the diagnosis and prognosis of AKI in patients with LC. The uNGAL level increases in LC patients with AKI [26], consistent with our results. uNGAL is a valuable tool for distinguishing acute tubular necrosis from pre-renal AKI or HRS and predicting mortality when combined with the MELD score in patients with LC [15].

Systemic inflammation contributes to the progression and development of complications in LC [27]. Moreover, it increases the serum levels of pro-inflammatory cytokines, including IL-6, IL-8, and tumor necrosis factor-alpha (TNF-α), in patients with LC [28]. Increased inflammatory mediators contribute to splanchnic arterial vasodilation and HRS development [29]. Among patients with decompensated LC, patients with HRS-AKI had higher levels of pro-inflammatory cytokines, including IL-6, IL-8, and TNF-α, than patients without AKI and presented decreased recovery from AKI and short-term mortality [30]. However, studies have demonstrated that inhibition of inflammation via NF-κB suppression could protect the kidney and liver in LT animal and cadmium-induced HRS animal models [31, 32].

sST2 has several features suitable for use as a biomarker, such as a short half-life and alteration in blood levels in response to disease progression or treatment [19]. Owing to these characteristics, ST2 is regarded as a potential biomarker that reflects the activity of various diseases. Studies have shown that an increase in the serum sST2 level is associated with an increase in disease activity and a poor prognosis. For instance, in a previous study, patients with ulcerative colitis or Crohn’s disease had higher serum sST2 levels than the negative controls, and the highest serum sST2 level was observed in patients with fistulizing Crohn’s disease [33]. In heart failure, increased serum sST2 level is associated with increased hospitalization and mortality [34]. Additionally, patients with AKI with a higher serum sST2 level demonstrate increased post-AKI mortality. The sST2 level increases according to the acute injury stage and shows no differences according to dialysis requirements [35]. Among patients who underwent CABG, increased sST2 concentration showed relationship with in-hospital mortality [36]. In acute on chronic liver failure patients with normal creatinine level, NGAL showed superiority on predicting 90 day mortality compared with cystain C [37]. Unfortunately, we could not analyze the association between sST2 or NGAL level and 90-day mortality because of the small sample size of the current study. We could gather data of only 87 LT recipients and 3 LT recipients who died within 90 days after LT.

However, an association between sST2 level and protective effects has been reported, which reflects our results regarding recovery from HRS-AKI. Among end-stage renal disease patients with hepatitis C virus infection, increased serum sST2 level results in less damage of the liver [38]. Several mechanisms have been proposed for the protective effects of sST2. Overexpression of sST2 exerts a protective effect against lipopolysaccharide (LPS)-induced acute lung injury by suppressing the production of pro-inflammatory cytokines by inhibiting NF-κB–DNA-binding activity in an animal model [39]. Another study using an in vitro LPS model showed that sST2 inhibits IκB degradation, which suppresses NF-κB–DNA binding, resulting in decreased production of the inflammatory cytokine IL-6 [40]. Another study reported that sST2 directly binds to immune cells, including macrophages, and inhibits the expression of pro-inflammatory cytokines, such as IL-6, IL-12, and TNF-α [41]. In a cisplatin-induced AKI animal model, the administration of IL-33, the primary ligand of ST2, induced elevated creatinine secretion, worsened acute tubular necrosis, and increased the apoptosis of tubular cells. The injection of sST2 showed a protective effect. sST2 antagonizes the IL-33/ST2L axis activity by acting as a decoy receptor [42]. Regarding the protective effect of sST2, increased sST2 level could be considered a negative feedback response to uncontrolled inflammatory reactions.

This study had several limitations. As this study was conducted in a single center, the sample size limited statistical power. Therefore, a multicenter study is needed to improve the statistical power and generalizability of the results. Additionally, our results showed a decrease in the serum sST2 level in non-recovered LTRs and LTRs with a history of RRT. In other words, the sST2 level decreased after severe disease progression or organ damage. In this regard, the serum sST2 level could reflect the status of active inflammation or active tissue destruction. Another limitation is that RRT may affect the serum sST2 level. To overcome this limitation, further analysis was performed. Our study included 10 HRS patients with stage 3 AKI. Among them, three patients underwent RRT before LT (all three patients underwent RRT using CRRT) and seven patients did not receive RRT. Furthermore, there was no difference in the serum sST2 level between the groups. All patients with HRS who underwent RRT before LT (n = 3) demonstrated normal kidney function 3 months post LT. Another study reported that the serum level of sST2 is not altered by hemodiafiltration [43], a newly developed dialysis technique. On the basis of these results, we thought that the serum sST2 level was not affected by RRT. However, we do not know the effect of CRRT on sST2 level. Further investigation is needed to explain the exact mechanism underlying the change in the sST2 level in extremely progressive diseases.

As the present study focused on a rare and specific topic such as the renal outcomes of LT in patients with HRS, the sample size was small. However, even under these unfavorable conditions, a significant difference in the serum sST2 levels after LT, which can be used to predict renal outcomes, was constantly observed. This finding suggests the potential of sST2 as a biomarker for renal recovery after LT in patients with HRS.

## Conclusions

Our results demonstrated that the serum sST2 level increased according to AKI or LC severity in patients with HRS-AKI. Both serum sST2 and uNGAL levels could be valuable markers for AKI in patients with LC. We also provided evidence that measuring sST2 level at the time of LT can be useful in predicting renal recovery and prognosis in LTRs with HRS-AKI.

## Supporting information

S1 Fig Elevated serum sST2 levels reflect deteriorated liver function according to multiple laboratory results. (TIF)
