# Supplementary figures and images for: Soluble suppression of tumorigenicity 2 is a potential predictor of post-liver transplant renal outcomes
Source: PLoS One. 2023 Nov 2;18(11):e0293844. doi: 10.1371/journal.pone.0293844 (PMC10621951; doi:10.1371/journal.pone.0293844)

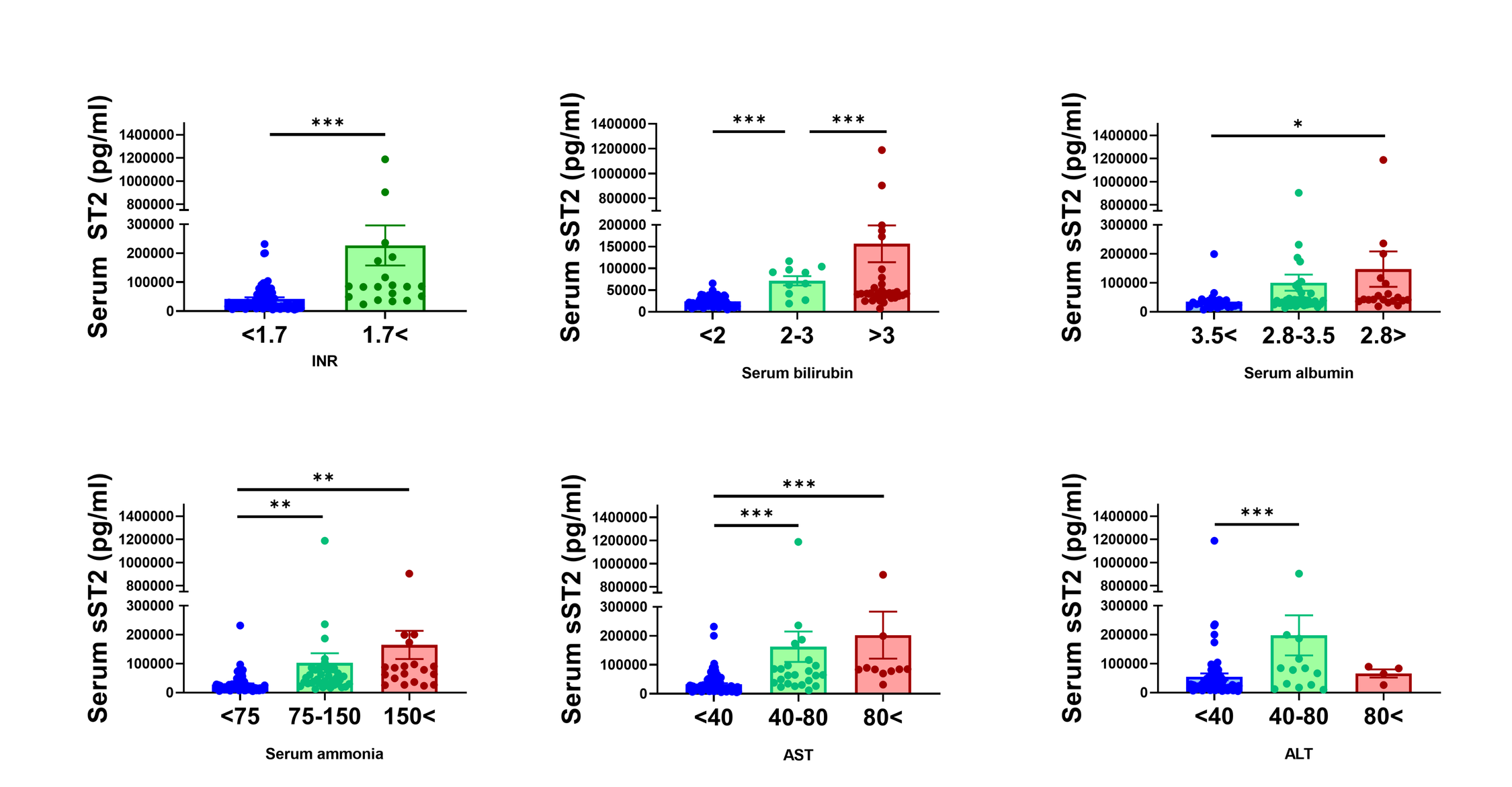

Supplement: S1 Fig — (TIF) [file pone.0293844.s001.tif]
